# Asymmetric expression of H19 and ADIPOQ in concave/convex paravertebral muscles is associated with severe adolescent idiopathic scoliosis

**DOI:** 10.1186/s10020-018-0049-y

**Published:** 2018-09-18

**Authors:** Heng Jiang, Fu Yang, Tao Lin, Wei Shao, Yichen Meng, Jun Ma, Ce Wang, Rui Gao, Xuhui Zhou

**Affiliations:** 1 Department of Orthopedics, Changzheng Hospital, Second Military Medical University, No.415 Fengyang Road, Shanghai, People’s Republic of China; 2 Department of Medical Genetics, Second Military Medical University, Shanghai, People’s Republic of China; 3 Shanghai Key Laboratory of Cell Engineering (14DZ2272300), Shanghai, People’s Republic of China

**Keywords:** Adolescent idiopathic scoliosis, Transcriptome, Paravertebral muscle, H19, ADIPOQ

## Abstract

**Background:**

Adolescent idiopathic scoliosis (AIS) is the most common paediatric spinal deformity. The etiology and pathology of AIS remain unexplained, and have been reported to involve a combination of genetic and epigenetic factors. Since paravertebral muscle imbalance plays an important role in the onset and progression of scoliosis, we aimed to investigate transcriptomic differences by RNA-seq and identify significantly differentially expressed transcripts in two sides of paravertebral muscle in AIS.

**Methods:**

RNA-seq was performed on 5 pairs of paravertebral muscle from 5 AIS patients. Significantly differentially expressed transcripts were validated by quantitative reverse polymerase chain reaction. Gene expression difference was correlated to clinical characteristics.

**Results:**

We demonstrated that ADIPOQ mRNA and H19 is significantly differentially expressed between two sides of paravertebral muscle, relatively specific in the context of AIS. Relatively low H19 and high ADIPOQ mRNA expression levels in concave-sided muscle are associated with larger spinal curve and earlier age at initiation. We identified miR-675-5p encoded by H19 as a mechanistic regulator of ADIPOQ expression in AIS. We demonstrated that significantly reduced CCCTC-binding factor (CCTF) occupancy in the imprinting control region (ICR) of the H19 gene in the concave-sided muscle contributes to down-regulated H19 expression.

**Conclusions:**

RNA-seq revealed transcriptomic differences between two sides of paravertebral muscle in AIS patients. Our findings imply that transcriptomic differences caused by epigenetic factors in affected individuals may account for the structural and functional imbalance of paravertebral muscle, which can expand our etiologic understanding of this disease.

**Electronic supplementary material:**

The online version of this article (10.1186/s10020-018-0049-y) contains supplementary material, which is available to authorized users.

## Background

Adolescent idiopathic scoliosis (AIS) is characterized by a three-dimensional deformity of the spine that occurs in the absence of underlying vertebral anomalies or obvious physiological defects (Altaf et al. 2013). The etiology and pathogenesis of AIS remain poorly explained, largely because of the genetic heterogeneity and lack of appropriate, tractable animal models (Boswell and Ciruna 2017). Genetic studies including traditional linkage analysis (Salehi et al. 2002), subsequent genome-wide association studies (Takahashi et al. 2011; Kou et al. 2013; Zhu et al. 2015; Sharma et al. 2011) and exome sequencing (Buchan et al. 2014; Haller et al. 2016) for AIS have identified more than 50 susceptible genetic variants, of which the function in AIS pathogenesis is yet undefined. On the other hand, numerous studies have suggested that some other factors, such as neuromuscular dysfunction (Wajchenberg et al. 2016; Grimes et al. 2016), and environment factors (Burwell et al. 2011) are associated with this disease.

The onset of scoliosis typically coincides with the adolescent growth spurt and the affected individuals are at a risk of increasing deformity until growth ceases. It has been reported that brace treatment significantly decreased the progression of high-risk curves, but still around 28% of AIS patients experienced exacerbation of scoliosis during or after bracing with undefined mechanism (Weinstein et al. 2013). Though several single nucleotide polymorphisms (SNPs) of certain genes such as neurotrophin-3 (Qiu et al. 2012; Ogura et al. 2017), and some parameters including the level of platelet calmodulin (Lowe et al. 2004) have been testified as predictors for spinal deformity progression in AIS, no method has been recommended for clinical use as diagnostic criteria (Noshchenko et al. 2015).

Functional and clinical assessments have correlated AIS with paravertebral muscle imbalance (Zapata et al. 2015; Wong 2015). Measurement of electromyography activity of the paravertebral muscles showed a higher amplitude of motor unit potentials on the convexity side (Stetkarova et al. 2016). In histological studies of multifidus muscles, increased proportion of type I fibers on the convex side and lower proportion of type I fibers on the concave side of the scoliotic curve have been reported (Stetkarova et al. 2016). Moreover, higher progression of AIS correlates significantly with the increased proportion of type I fibers on the convex side (Stetkarova et al. 2016). We have previously demonstrated that the muscle volumetric and fatty infiltration imbalance occur in all of the levels of the vertebrae involved in the major curve of AIS with larger muscle volume on the convex side and higher fatty infiltration rate in the concave side (Jiang et al. 2017). Some studies have uncovered asymmetric expression of melatonin receptor (Wong 2015) and transforming growth factor-beta signaling (Nowak et al. 2014) in bilateral paravertebral muscles of AIS. There are also some interesting findings that some AIS risk loci identified by genetic study located in regions near or within genes associated with muscle biogenesis (Sharma et al. 2015a). These evidences indicated that paravertebral muscle might play an important role in the initiation and progression of AIS. Nonetheless, the mechanism of the complex muscle tissue changes remains unclear. This study employed RNA-seq to evaluate whole transcriptomic changes in the concave- and convex-sided paravertebral muscle at the apex level of the main curve in patients with AIS. And our data identified differential expression of H19 and ADIPOQ mRNA between the two sides of paravertebral muscle in AIS patients. More importantly, lower expression of H19 and higher expression of ADIPOQ mRNA in concave-sided muscle correlate positively with curve severity and age at initiation.

## Methods

### Patients

The research project was approved by the ethics department of Shanghai Changzheng Hospital, Shanghai. We have consensus with all participants. All the procedures were done under the Declaration of Helsinki and relevant policies in China.

We collected 5 pairs of paravertebral muscle samples from 5 AIS patients during spinal surgery (mean age 14.20 ± 1.92 yrs.; female; *n* = 5) for RNA-seq. Paravertebral muscle samples were obtained from both sides of multifidus muscle of AIS patients at the apex level of the main curve. Muscle tissues were stored in liquid nitrogen immediately. To validate the differentially expressed transcripts identified by RNA-seq, 60 pairs of paravertebral muscle tissues were obtained from different patients with AIS during surgery. AIS was diagnosed and classified based on the Lenke classification. The clinical characteristics of the enrolled patients were summarized in Additional file 1: Table S1. Samples of patients with congenital scoliosis (CS) were collected from both sides of multifidus muscle at the apex level of the curve at surgery in 25 age-matched individuals during spinal surgery. CS patients enrolled in this study had major thoracic curves and similar magnitudes of curves compared with those of AIS patients (54.48 ± 10.09 vs. 59.32 ± 15.43, *p* = 0.09, Additional file 1: Figure S1). And 16 age-matched patients undergoing spinal surgery for thoracic spinal fracture were enrolled as non-AIS group and the multifidus muscle samples were obtained at the upper level of the instrumented vertebra.

### Total RNA extraction and RNA-seq

Total RNA was isolated from fresh-frozen tissue samples and extracted using TRIzol reagent (Invitrogen, Carlsbad, CA, USA). Total RNA quality and quantity were determined using a Nanodrop 8000 UV-Vis spectrometer (Thermo Scientific Inc., Waltham, MA, USA). RNA-seq was performed using total RNA samples with a quantity greater than 10 μg and an RNA integrity number > 6.0. If the quantity of total RNA was less than 10 μg or the RNA integrity number was less than 6.0, another muscle sample was used for RNA isolation. Library construction for whole transcriptome sequencing was performed using the Truseq RNA sample preparation v2 kit (Illumina, San Diego, CA, USA) as described previously (Hong et al. 2017).

### Quantitative real-time polymerase chain reaction (qRT-PCR)

Total RNA was extracted and then reverse-transcribed into cDNA using the cDNA Reverse Transcription Kit from Applied Biosystems (Foster City, CA, USA). qRT-PCR was conducted using SYBR Green Master Mix on the ABI 7900HT fast real-time PCR System (Applied Biosystems, Waltham, MA, USA). The following thermal settings were used: 95 °C for 10 min followed by 40 cycles of 95 °C for 15 s and 60 °C for 1 min. The primers used for H19, miR-675-5p, PCK1, FABP4, SCD, PLIN1, ADIPOQ, U6 (internal control for miRNAs), and 18 s (internal control for mRNAs and lncRNAs) are listed in Additional file 1: Table S2. The ∆∆Ct method was used to calculate the relative expression level of mRNAs or miRNAs on both sides and fold change was presented related to convex side.

### Cell culture

Human skeletal muscle satellite cells (HSkMSC) were obtained from ZhongQiaoXinZhou Biotech Co. (Shanghai, China) and cultured at sub-confluent density in growth medium consisting of Ham’s F-10 supplemented with 20% fetal bovine serum (FBS) and 1% antibiotics (PeSt). All cell-based in vitro experiments were repeated in triplicate. To initiate differentiation into myotubes, Ham’s F-10/20% FBS was removed from cells and Dulbecco’s modified Eagle’s medium (DMEM) containing 1% PeSt/4% FBS was added for 48 h. After this, medium was changed to DMEM containing 1% PeSt/2% FBS. Differential medium was changed every 3 days, and cells were harvested at the indicated times. The HEK293T cells were obtained from Cellbank (SIBS, Shanghai, China) and cultured in DMEM with 10% FBS and 1% antibiotics.

### RNA oligoribonucleotides and transient transfection

A chemically-modified double-stranded miR-675-5p mimic and the corresponding miRNA mimic control (mimic NC) were obtained from RiboBio Co. (Guangzhou, China). Inhibitor of miR-675-5p and the corresponding inhibitor control (inhibitor NC) were from RiboBio Co. (Guangzhou, China). The sequences are listed in Additional file 1: Table S2. Cells at 70–80% confluence were transfected with miRNA mimics, or inhibitors using Lipofectamine 2000 (Invitrogen, Carlsbad, CA, USA) according to the manufacturer’s instructions as described previously (Dey et al. 2014).

### Dual luciferase reporter assay

The 3′-untranslated region (3’UTR) of ADIPOQ was amplified with 5’-GCCTCCTGAATTTATTATTGTTC-3′ and 5′-GTT CGG TGT GGT AGA CCG A-3′, and subcloned in psiCheck-2 (Promega, Madison, WI, USA). As specificity control, the seed sequence GGCTC of miR-675-5p target sites on the 3’UTR of ADIPOQ was replaced by GGGGG using site-directed mutagenesis. HEK293T cells grown in 48-well plates were transfected with 100 nM miR-675 mimic or control, 40 ng luciferase reporter, and 4 ng pRL-TK, plasmid expressing Renilla luciferase (Promega) using Lipofectamine 2000 (Invitrogen). The Renilla/firefly luciferase activities were measured 24 h after transfection using the Dual Luciferase Reporter Assay System (Promega). All luciferase values were normalized to those of Renilla luciferase and expressed as fold-induction relative to the basal activity.

### Western blotting

Cells were harvested, washed with PBS, and lysed in RIPA buffer. Proteins were separated by 12% sodium dodecyl sulfate–polyacrylamide gel electrophoresis and transferred to polyvinylidene fluoride membranes. Primary antibodies against ADIPOQ (Abcam, Cambridge, UK) and beta-actin (Cell Signaling Technology,Bevereverly, MA, USA) were diluted 1:1,000. The intensities of the bands obtained by Western blotting analysis were quantified using ImageJ software (http://rsb.info.nih.gov/ij/). The background was subtracted, and the signal of each target band was normalized to that of the beta-actin band.

### Chromatin immunoprecipitation (ChIP) assay

ChIP assays were performed using the SimpleChIP Plus Enzymatic Chromatin IP kit (Cell Signaling Technology) according to the manufacturer’s instructions. When harvesting tissue, unwanted material such as fat and necrotic material was removed. Then the tissue was cross-linked with 1.5% formaldehyde for 20 min. Nuclei preparation and chromatin digestion was performed by micrococcal nuclease. Chromatin was sonicated on ice to generate chromatin fragments of 150–900 bp. Antibodies against CTCF (Cell Signaling Technology) or negative control rabbit IgG (Cell Signaling Technology) were used in chromatin immunoprecipitation. Then chromatin was eluted from antibody/protein G magnetic beads and reverse cross-linked. Input control DNA or immunoprecipitated DNA was quantified by standard PCR method, using SimpleChIP Human H19/IGF2 ICR Primers (Cell Signaling Technology). The following thermal settings were used: 95 °C for 5 min followed by 34 cycles of 95 °C for 30s, 62 °C for 30s and 72 °C for 30s, then 72 °C for 5 min. PCR product was analyzed by 10% polyacrylamide gel electrophoresis and the signal of each target band was quantified using ImageJ software mentioned above.

### Statistical analysis

Statistical analyses were performed using SPSS version 16.0 (SPSS, Chicago, IL, USA). All data are expressed as mean ± standard deviation (SD). Differences between groups were analyzed using Student’s t-test. In cases of multiple-group testing, one-way analysis of variance was conducted. Pearson correlation test was used to analyze the correlation between the expression difference of genes and the clinical parameters. A two-tailed value of *p* < 0.05 was considered statistically significant.

## Results

### Transcriptomic alterations in the concave and convex side of paravertebral muscle of AIS patients

**Fig. 1:**
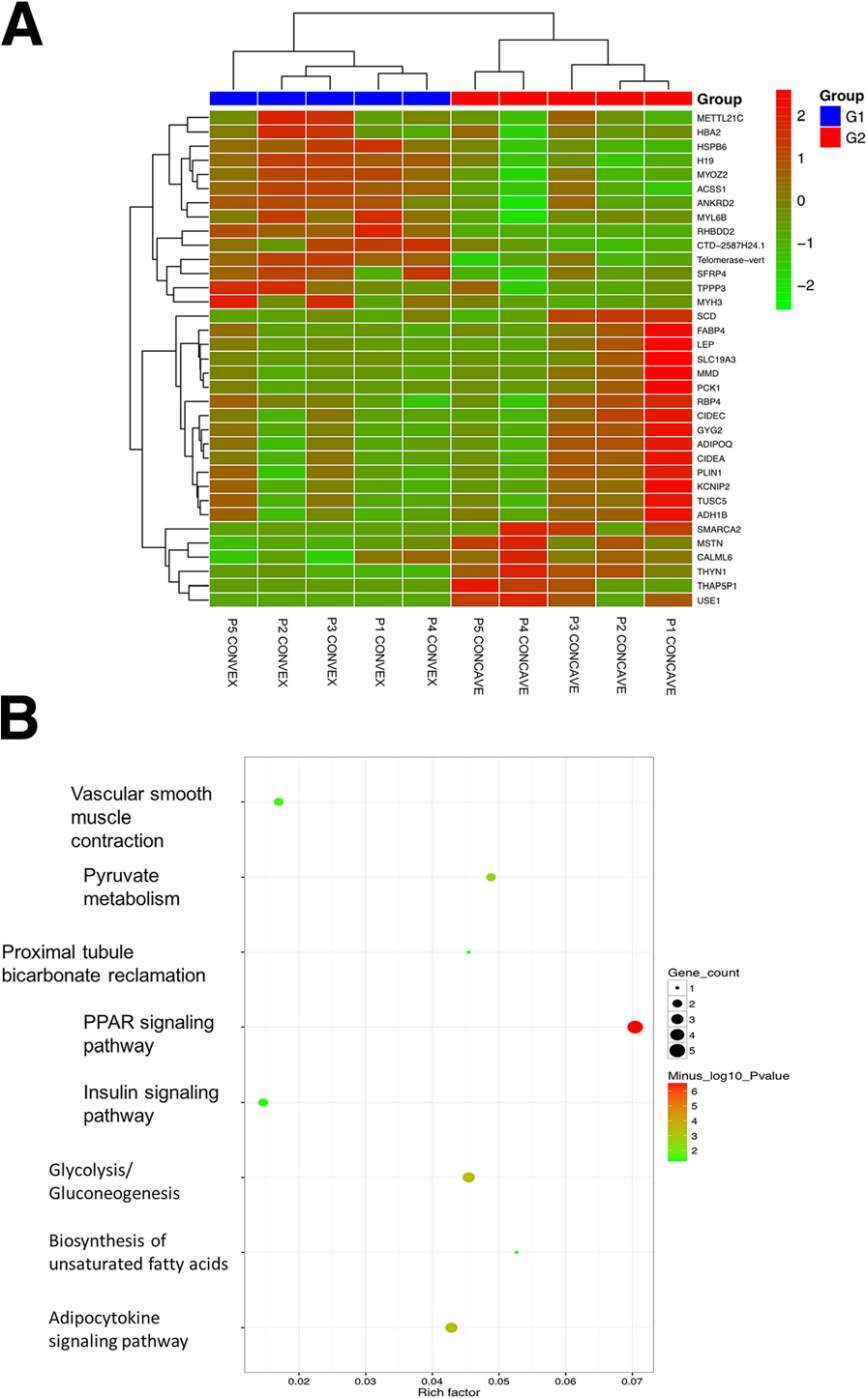
Differentially expressed genes in convex/concave paravertebral muscles in AIS. **a** Heat map and hierarchical tree comparing differences in gene expression in convex-sided (G1) and concave-sided (G2) paravertebral muscle of AIS patients (*n* = 5). Each line represents the changes (direction and intensity) in each transcript, and each column represents an individual sample according to group (P1CONCAVE represents the concave sided muscle sample of patient 1 and P1CONVEX represents the convex sided muscle sample of patient 1). The lines represent transcripts altering between two sides (*p* < 0.05 and fold change> 2). **b** KEGG enrichment analyses of differentially expressed genes (DEGs). The prominently over-represented pathways included those for PPAR signaling pathway and biosynthesis of unsaturated fatty acids

RNA-seq was performed for 5 pairs of paravertebral muscle tissues from 5 AIS patients. Differentially expressed genes (DEG) analysis using the R-based DESeq package identified a total of 40 genes differentially expressed between the convex and concave side of paravertebral muscles. The gene expression profiles of the sample were visualized and compared using the heat maps (Fig. 1a). DEGs (showed in Additional file 1: Table S3) in two sides of paravertebral muscles of AIS patients clustered together and there were 16 genes higher expressed in the convex side relative to the concave side.

To understand the biological meaning of DEGs, KEGG enrichment analysis of DEGs were conducted. The prominently over-represented pathways included those for peroxisome proliferator-activated receptor (PPAR) signaling pathway (fold enrichment = 31.5; *p* = 3.01E-07; FDR = 1.38E-05), glycolysis/gluconeogenesis (fold enrichment = 20.3; *p* = 3.75E-04; FDR = 6.84E-03), adipocytokine signaling pathway (fold enrichment = 19.2; *p* = 4.46E-04; FDR = 6.84E-03), pyruvate metabolism (fold enrichment = 21.8; *p* = 3.62E-03; FDR = 4.17E-02) and vascular smooth muscle contraction (fold enrichment = 7.6; *p* = 2.77E-02; FDR = 2.54E-01) (Fig. 1b). It is interesting to note that several genes related with PPAR signaling pathway, such as PCK1, FABP4, SCD, PLIN1, ADIPOQ, MSTN, showed a higher expression in the concave side of the paravertebral muscles, since PPAR signaling pathway plays a major regulatory role in postnatal myogenesis and muscle fiber type (Wang et al. 2004; Chandrashekar et al. 2015).

### Significantly differentially expressed transcripts between two sides of paravertebral muscle of AIS patients

**Fig. 2:**
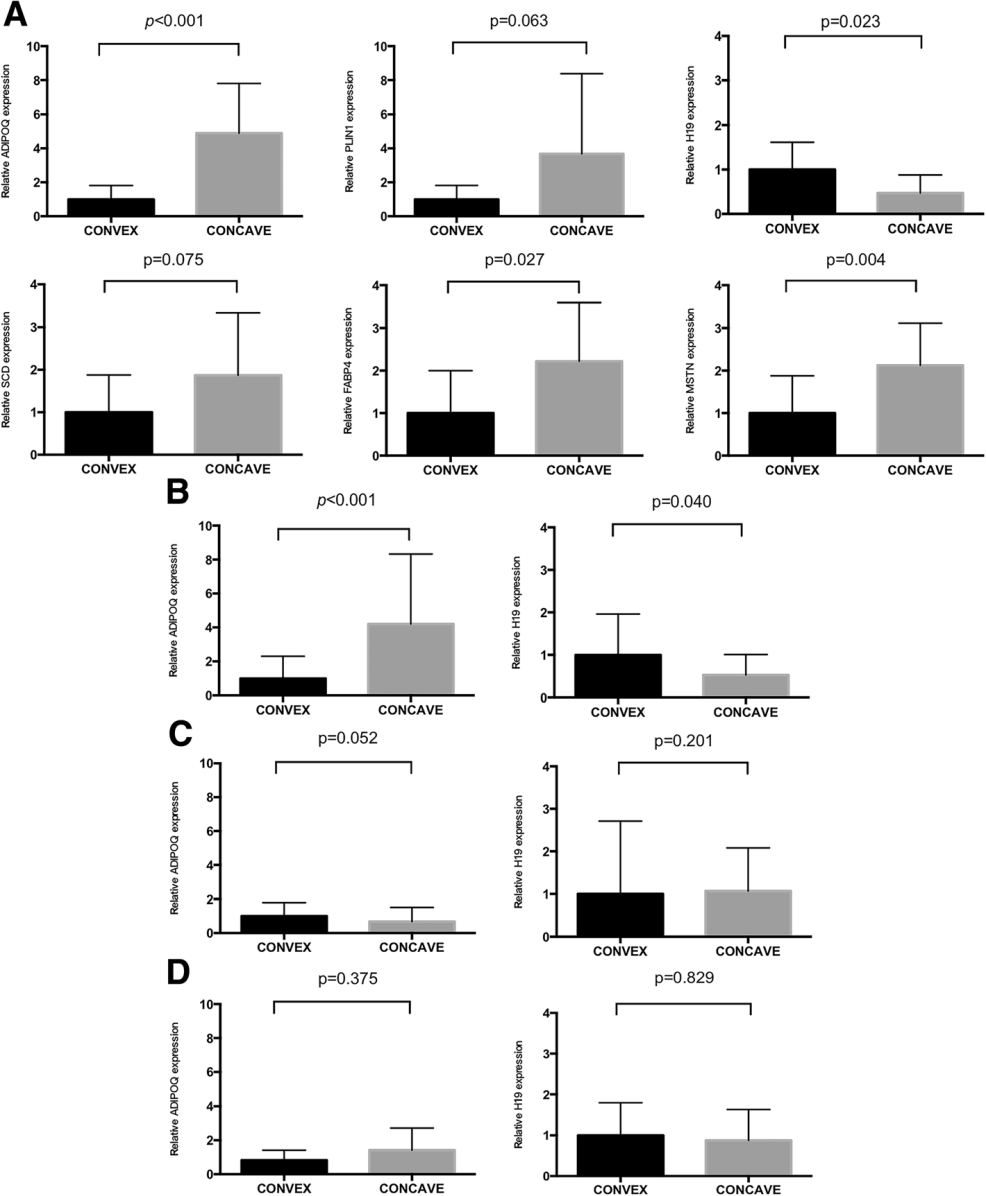
H19 and ADIPOQ mRNA is significantly differentially expressed between two sides of paravertebral muscle of AIS patients. Paravertebral muscles were obtained during the fusion surgery. Tissue samples were processed for assessment of expression of mRNA of PPAR signaling pathway related genes (SCD, FABP4, ADIPOQ, PLIN1, MSTN) and H19, as described in Materials and Methods. For (**a**) AIS patients (*n* = 10), the mRNA of ADIPOQ, FABP4, MSTN genes and H19 showed significantly differential expression in concave/convex muscle tissues. For (**b**) AIS patients (*n* = 50), ADIPOQ mRNA and H19 are still significantly differentially expressed between two sides of muscle tissues. For (**c**) age-matched CS group (*n* = 25), ADIPOQ mRNA or H19 expression showed no significant difference between convex and concave sided muscle tissue. For (**d**) age-matched non-AIS group (*n* = 16), ADIPOQ mRNA or H19 expression showed no significant difference between right and left sided muscle tissue. Results are expressed as mean ± SEM, and the relative expression of the studied genes are represented as fold change related to the convex side

To test the quality of RNA-seq, we screened for the expression of 5 PPAR signaling pathway related genes (FABP4, SCD, PLIN1, ADIPOQ, MSTN, the expression of PCK1 was not quantified as its expression level was too low to be detected by qPCR) using qPCR in a series of 10 pairs of muscle samples from AIS patients. In addition, H19, a long noncoding RNA functioning in skeletal muscle differentiation and regeneration (Dey et al. 2014), which showed a differential expression in RNA-seq results, was also included. At first, we investigated the expression of specific markers of adipocytes (PPARγ, C/EBPα) and myocytes (Myog, MHC) to ensure a similar muscle tissue rate biopsy in each side (Additional file 1: Figure S2). The mRNA of ADIPOQ, FABP4, and MSTN genes showed significantly higher expressions (*p* < 0.001, 0.027, 0.004, respectively), while H19 showed significantly lower expression in concave-sided muscle tissue (*p* = 0.023) (Fig. 2a) when compared to convex-side.

Since the expression of ADIPOQ mRNA and H19 showed the most significant difference and recent studies have uncovered that they both play fundamental roles in regulation of muscle metabolic and contractile function (Dey et al. 2014; Iwabu et al. 2010), we measured ADIPOQ mRNA and H19 expressions in a second AIS cohort of 50 pairs of muscle tissues to validate the differential expression. Both genes showed significantly differential expression (ADIPOQ, *p* < 0.001; H19, *p* = 0.040) in two sides of the paravertebral muscles (Fig. 2b).

To verify whether the mRNA of ADIPOQ and H19 are also dysregulated in patients with congenital scoliosis (CS), we assessed ADIPOQ mRNA and H19 expression in 25 paired muscle samples of CS. Neither ADIPOQ mRNA nor H19 expression showed significant differences between convex and concave sided muscle tissue (*p* = 0.052 and 0.201, respectively) (Fig. 2c).

To further ascertain whether this differential expression was specifically present in AIS patients, we analyzed ADIPOQ mRNA and H19 expression in muscle tissues obtained from a series of 16 age-matched patients undergoing spinal surgery for thoracic spinal fracture. Neither ADIPOQ mRNA nor H19 expression showed significant differences between right and left sided muscle tissue (*p* = 0.375 and 0.829 respectively) (Fig. 2d).

### ADIPOQ and H19 expression in patients with AIS is associated with curve severity and age at initiation

**Fig. 3:**
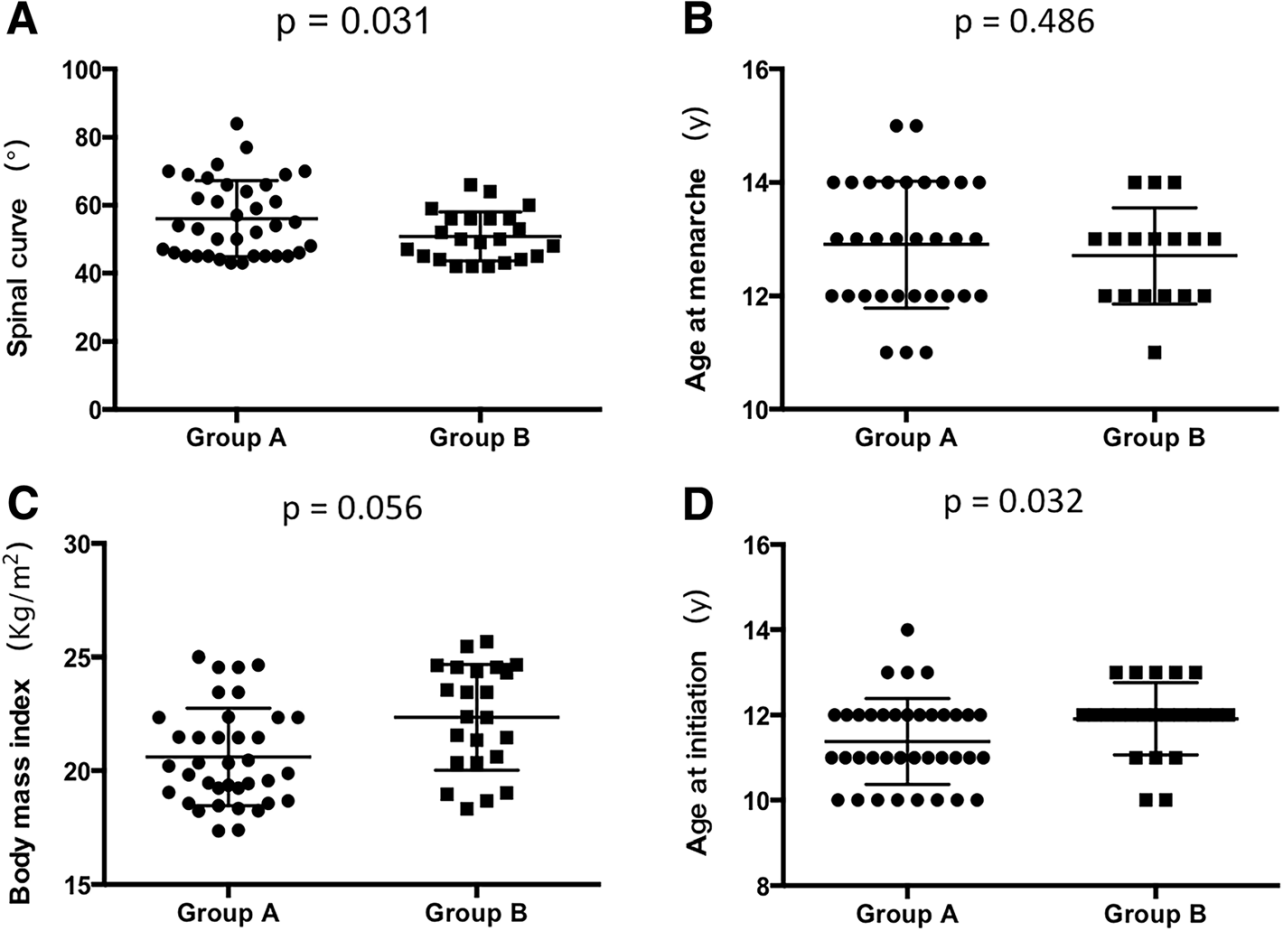
ADIPOQ mRNA and H19 expression pattern in paravertebral muscles of AIS patients is associated with curve severity and age at initiation. Patients with lower H19 and higher ADIPOQ mRNA expression levels in concave-sided muscles were included in Group A (*n* = 37), and the remained patients were included in group B (*n* = 23). For (**a**), Patients in Group A showed a bigger spinal curve (*p* = 0.031) compared with patients in Group B. For (**b**), Group A and Group B showed no difference in age at menarche (*p* = 0.486). For (**c**), Patients in Group A tended to have a declined body mass index (BMI) (*p* = 0.056). For (D), Patients in Group A (*n* = 37) showed an earlier age at initiation (*p* = 0.032) compared with patients in Group B (*n* = 23). Results are expressed as mean ± SEM

Then we were interested in comparing features of clinical characteristics, such as the magnitude of spinal curve, age at menarche, body mass index and age at initiation, between different samples with different ADIPOQ and H19 expression patterns. We classified the enrolled 60 AIS patients studied in Fig. 2 as group A and group B. In group A, the expression level of H19 was lower and ADIPOQ mRNA higher in concave-sided muscle tissues compared with the corresponding convex side (low H19/high ADIPOQ). And the remained patients were included in group B. Patients in group A showed a larger magnitude of spinal curve (*p* = 0.031) and earlier age at initiation (*p* = 0.032) compared with that of group B. And patients in group A tend to have a decreased BMI, but the difference is not significant (*p* = 0.056) (Fig. 3).

**Fig. 4:**
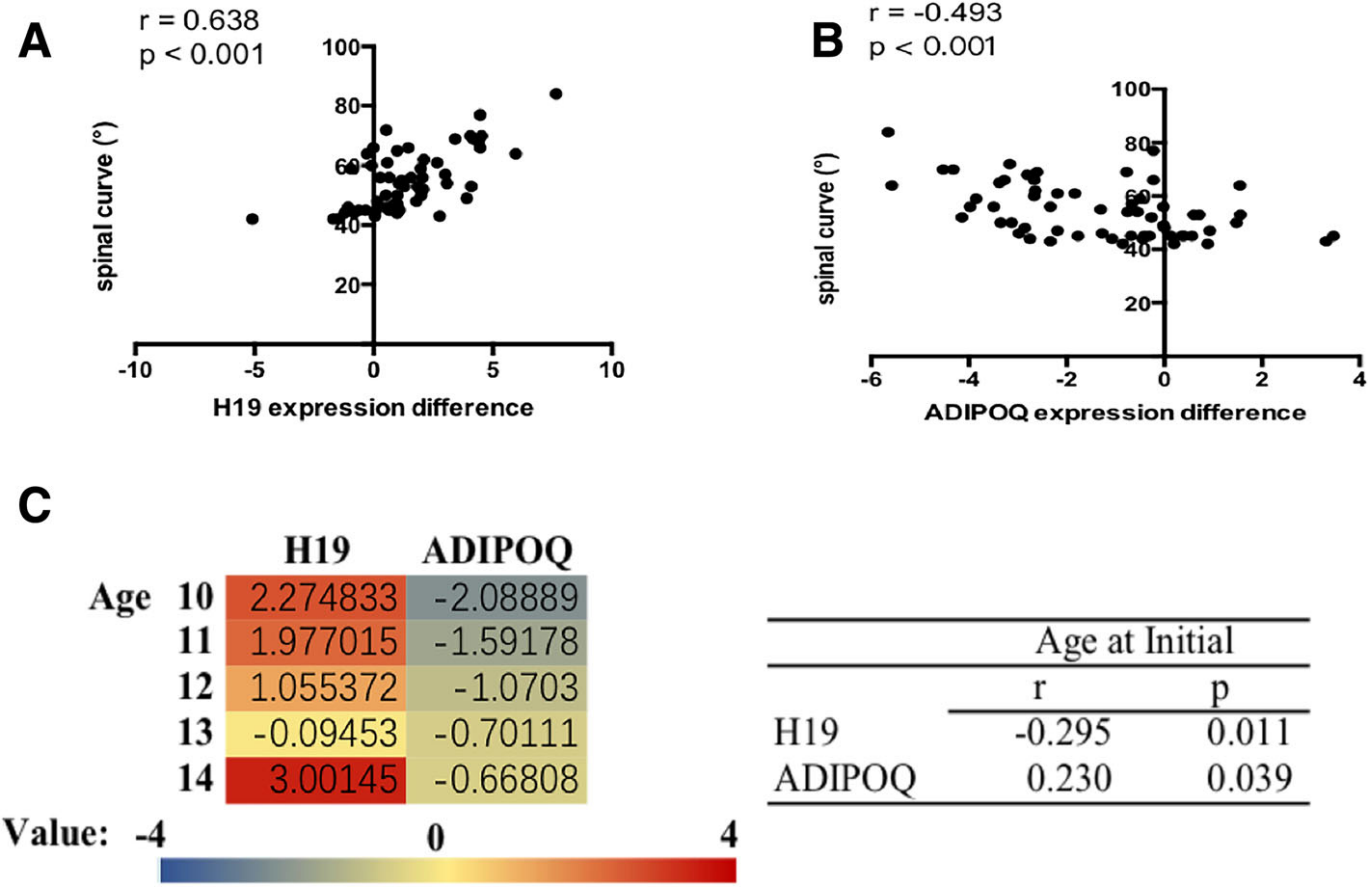
Expression difference of H19 and ADIPOQ mRNA in paravertebral muscles of AIS patients is associated with curve severity and age at initiation. The relative expression difference of studied genes was calculated as the relative expression level of one gene in concave sided muscle minus that in convex sided. For (**a**), correlation analysis identified that the relative expression difference of H19 in AIS patients (*n* = 60) was positively correlated with the magnitude of spinal curve. For (**b**), the relative expression difference of ADIPOQ mRNA in AIS patients (*n* = 60) was negatively correlated with the magnitude of spinal curve. For (**c**), Heat map showed significant correlation of the expression difference of H19 and ADIPOQ mRNA in AIS patients (*n* = 60) with age at initiation of AIS

We also performed correlation analysis to identify whether gene expression difference between two sides of paravertebral muscles was related to these clinical parameters. The relative expression difference of H19 (concave-convex) was significantly correlated with Cobb’s angle (*r* = 0.638, *p* < 0.001) and age at initiation (*r* = − 0.295, *p* = 0.011) (Fig. 4a-c), and the relative expression difference of ADPOQ mRNA (concave-convex) was also significantly correlated with spinal curve (*r* = − 0.4926, *p* < 0.001) and age at initiation (*r* = 0.230, *p* = 0.039) (Fig. 4b, c). The expression difference of H19 or ADIPOQ mRNA showed no significant correlation with patients’ age at menarche or BMI (Additional file 1: Figure S3).

### H19 regulated the expression of ADIPOQ by miR-675-5p

**Fig. 5:**
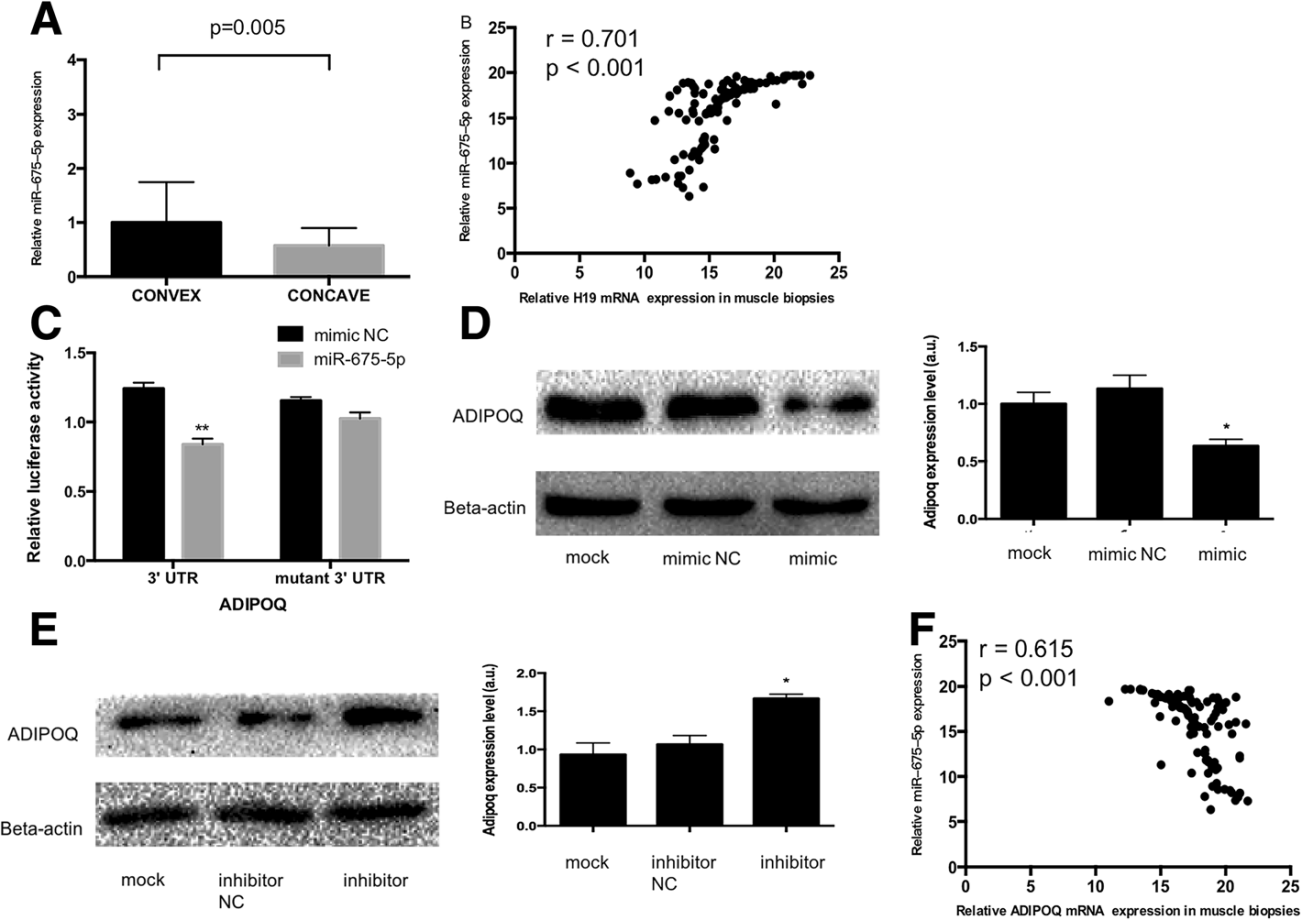
miR-675-5p directly targeted the 3’ UTR of ADIPOQ. **a** Tissue samples were processed for assessment of expression of miR-675-5p as described in Materials and Methods. miR-675-5p showed a significantly higher expression in convex-sided muscle tissue in AIS patients (*n* = 60). Results are expressed as mean ± SEM, and the relative expression of miR-675-5p is represented as fold change related to the convex side. **b** Correlation analysis identified that the expression level of miR-675-5p in muscle tissues was positively correlated with that of H19 in AIS patients (*n* = 60). **c** HEK293T cells were co-transfected with miR-675-5p mimics/mimic NC and plasmid with wild type or mutant luc-3’UTR of ADIPOQ. Luciferase values were normalized to those of Renilla luciferase and expressed as fold-induction relative to the basal activity. Co-transfection of luc-3’UTR but not mutant miR-675-5p luc-3’UTR with a miR-675-5p mimic significantly impairs luciferase expression in HEK293T cells. The experiments were performed three times. Results are expressed as mean ± SEM. **, *P* < 0.01 versus luc-3’UTR. For (**d**, **e**), HSkMSC cells were subjected to transfection and differentiation induction as indicated. After 72 h, total cellular proteins were extracted from harvested cells. Levels of ADIPOQ protein were assessed with Western blot using anti-ADIPOQ antibody, as described in Materials and Methods. Left panels illustrate the autoradiographs of representative immunoblots; right panels show densitometric analysis of the immunoblot data (normalized to beta-actin). The experiments were performed three times. Results are expressed as mean ± SEM. *, *P* < 0.05 versus NC/Mock. **f** Correlation analysis identified that the expression level of miR-675-5p in muscle tissue samples was negatively correlated with that of ADIPOQ mRNA in AIS patients (*n* = 60)

H19 is a long non-coding RNA of which transcription persists only in skeletal muscle in adult, and functions as a primary miRNA precursor for microRNA-675 (miR-675) which generates two mature miRNAs, namely miR-675-5p and miR-675-3p (Lewis et al. 2016). MiR-675-5p and miR-675-3p mediate the regulatory function of H19 in skeletal muscle differentiation (Dey et al. 2014). So, we quantified expression of miR-675-5p and miR-675-3p using qRT-PCR in AIS patients and identified that both of two miRNA showed significantly higher expressions in convex-sided muscle tissue (Fig. 5a, Additional file 1: Figure S4A). Moreover, we demonstrated that the expression levels of miR-675-5p and miR-675-3p in muscle tissue were positively correlated with that of H19 (*r* = 0.701, *p* < 0.001; *r* = 0.689, *p* < 0.001, respectively) (Fig. 5b, Additional file 1: Figure S4B).

The miRNA machinery is a regulator of gene expression and we assumed that this kind of epigenetic regulation was involved in the imbalanced ADIPOQ expression as the expression difference of ADIPOQ mRNA represented an opposite direction compared with H19. We performed bioinformatics algorithms using RNA22 software scanning for miRNAs potentially targeting ADIPOQ mRNA (Miranda et al. 2006). The transcript of ADIPOQ was found to contain several putative miR-675-5p-binding sites but not miR-675-3p (Additional file 1: Table S4). Among these, miR-675-5p possessed the maximum likelihood of binding to the 3’UTR of ADIPOQ (3403–3428 nt) (∆G = − 17.70Kcal/mol). Thus, we chose this target site and subcoloned the 3’UTR of ADIPOQ mRNA in the Renillaluciferase expression cassette (Luc-3’UTR) of a luciferase reporter construct. The ectopic overexpression of miR-675-5p significantly (*p* < 0.0001) inhibited luciferase activity in the ADIPOQ construct, while mutation of the miR-675-5p-binding site abolished the inhibitory effect of miR-675-5p on ADIPOQ reporter activity (Fig. 5c). Then we assessed the expression of ADIPOQ in HSMCs overexpressing miR-675-5p. Compared with the negative control, miR-675-5p substantially reduced the protein levels of ADIPOQ during HSMC differentiation (Fig. 5d). In a reciprocal experiment, we inhibited the endogenous miR-675-5p using antisense inhibitor and observed increased levels of ADIPOQ (Fig. 5e). These data demonstrate that miR-675-5p can silence ADIPOQ expression, which is also reflected in vivo where a significant (*r* = − 0.615, *p* < 0.001, Fig. 5f) inverse correlation of ADIPOQ mRNA and miR-675-5p expression in muscle samples studied in Fig. 2.

### H19 expression was regulated by CTCF occupancy in the H19 imprinting control region (ICR)

H19 is an imprinted gene, which is abundantly expressed maternally in embryonic tissues but is strongly repressed after birth (Dey et al. 2014; Keniry et al. 2012; Gabory et al. 2010). The H19- insulin like growth factor (IGF2) gene locus contains differentially methylated regions that control parent-of-origin expression. It is reported that epigenetic-related dysregulation of H19 may play complex roles in different disorders, including stenotic aortic valves disease (Hadji et al. 2016). Thus, we profiled both sides of paravertebral muscles for the level of CpG methylation in the promoter region of H19 (~ − 900 bp from the transcription start site) and found no differences between two sides (Additional file 1: Figure S6).

**Fig. 6:**
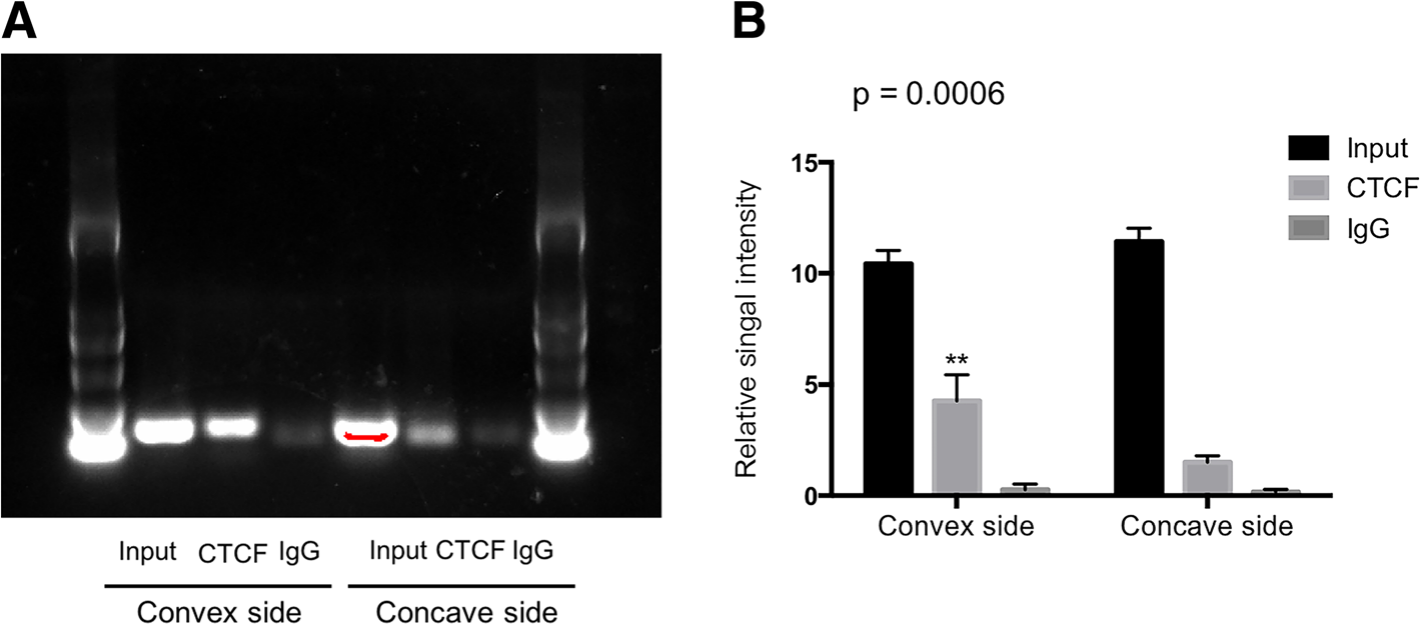
H19 expression was regulated by CTCF occupancy in the H19 imprinting control region. **a**, **b** Paravertebral muscles were harvested from AIS patients during the fusion surgery. The tissues were subjected to Nuclei preparation and chromatin digestion, and the CTCF-binding status at the H19 ICR were assessed with CHIP assay, as described in Materials and Methods. **a** represent electrophoretogram of PCR product by 10% polyacrylamide gel electrophoresis. **b** CTCF occupancy in the H19 ICR showed significantly reduced occupancy in the concave-sided muscle tissue samples. The experiments were performed three times. Results are expressed as mean ± SEM. *n* = 4 in each group. **, *P* < 0.01 versus concave side

The CTCF protein binding to the ICR of the H19 gene promotes enhancer function at the H19 prompter, which is another important mechanism controlling H19 expression (Ideraabdullah et al. 2011; Phillips and Corces 2009). To determine the differences of the CTCF-binding status at the H19 ICR, we analyzed the levels of CTCF occupancy in this region (~ − 4 k bp from the transcription start site) in both sides of paravertebral muscles using CHIP assay. The occupancy of CTCF protein in the H19 ICR significantly decreased in the concave-sided muscle tissue (Fig. 6).

## Discussion

AIS is a common and unexplained spinal deformity in children and poses an increasing burden on global health. The phenotypic heterogeneity of AIS patients makes it difficult to form any efficient strategy to prevent this disease or its progression (Altaf et al. 2013). One of the biggest challenges in etiologic research of AIS is that the affected individuals present no obvious structural deficiencies or pathologic changes in the vertebral column and associated soft tissues, expect the curvation of the spine (Weinstein and Dolan 2015). So numerous theories have been put forward to define the disease mechanisms, including genetic predisposition (Gorman et al. 2012), neuromuscular dysfunction (Wajchenberg et al. 2016), and environment factors (Burwell et al. 2011). The fact that scoliosis is frequently seen as a part of the phenotypic spectrum of heritable syndromes, in particular, disorders of neuromuscular development, also serves as a reminder of the importance of the muscle imbalance in AIS pathogenesis (Hresko 2013). By studying the differentially expressed transcripts between two sides of paravertebral muscle of AIS patients, we aimed to identify drivers or key molecules for AIS-associated paravertebral muscle changes.

Here, we demonstrate that H19 shows a higher expression in convex-sided muscle and ADIPOQ higher expression in concave side. H19 is a paternally-imprinted gene that does not encode a protein, but rather a 2.3-Kb ncRNA (Borensztein et al. 2013). It is reported that H19 can promote skeletal muscle differentiation and regeneration (Dey et al. 2014), and regulate glucose metabolism in muscle cells as well (Gao et al. 2014). Adiponectin (ApN) encoded by ADIPOQ gene, is a circulating hormone and it has been demonstrated that during skeletal muscle differentiation, myotubes represent an autocrine system for ApN production (Fiaschi et al. 2009). In addition, low ADIPOQ expression associates with degenerative muscle disease, such as Duchenne muscular dystrophy (Abou-Samra et al. 2015). Here, we demonstrate for the first time that H19 and ADIPOQ expressions are inconsistent in two sides of the paravertebral muscle in AIS patients.

Our data demonstrate that lower H19 levels and higher ADIPOQ levels in concave-sided muscle tissues significantly correlate with larger spinal curve and earlier age at initiation. Moreover, our data demonstrate that the relative differential expression of H19 and ADIPOQ (concave-convex) significantly correlate with spinal curve and age at initiation. But we didn’t find asymmetric expression of H19 or ADIPOQ between two sides of paravertebral muscles of age-matched CS patients or non-scoliotic controls. Taken together, the evidence suggests that an asymmetric expression of H19 and ADIPOQ is a relatively specific event in AIS disease progression.

In this study, we identified imbalanced-expressed H19 regulatory mechanism that induces ADIPOQ differential expression in two sides of paravertebral muscles in AIS. We demonstrate that ADIPOQ mRNA is targeted by miR-675-5p, a miRNA encoded by H19. Consistent with this, the expression levels of miR-675-5p in muscle tissues positively correlate with that of H19 and negatively correlate with that of ADIPOQ. H19-derived miR-675-5p has been reported to directly target DNA replication initiation factor Cdc6 to promote skeletal muscle differentiation (Dey et al. 2014) and inhibit adipocyte differentiation of mesenchymal stem cells through down-regulating HDACs (Huang et al. 2016). Our data demonstrate that ADIPOQ is a novel target gene of miR-675-5p.

Muscle derived ApN has been reported to participate in the activation of autophagy, which is required for muscle differentiation (Gamberi et al. 2016). In skeletal muscle, ApN exerts its metabolic effects through adiponectin receptor1 (AdipoR1) (Delaigle et al. 2004). ApN has been suggested as a main effector in the regulation of muscle lipid metabolism by stimulating fatty-acid oxidation (Yamauchi et al. 2002). Thus dysregulated ADIPOQ expression may contribute to the fatty infiltration imbalance of paravertebral muscle in AIS (Additional file 1: Figure S7) (Jiang et al. 2017). ApN has also been reported to modulate oxidative stress-induced mitophagy and alleviate skeletal muscle inflammation through miR-711 (Boursereau et al. 2017). In consideration of oxidative stress and local inflammation have been associated with muscle dysfunction (Acharyya et al. 2007; Powers et al. 2012), the lower ADIPOQ levels in the convex-sided muscle may contribute to the paravertebral muscle weakness in convex side (Martinez-Llorens et al. 2010). On the other hand, ApN has been reported to increase mitochondria and oxidative myofiber in skeletal muscle through activation of CaMKK, AMPK, SIRT1, which were correlated with increased type I myofiber, insulin resistance and exercise endurance and supported the view of stronger muscle contraction of concave-sided paravertebral muscle in AIS (Iwabu et al. 2010; Abou-Samra et al. 2015). The existence of an increased proportion of type I fibers on the convex-sided paravertebral muscles in AIS suggests the existence of additional regulatory mechanisms of myofiber identity and muscle performance.

Epigenetic mechanisms, including DNA methylation and transcription regulator CTCF have been reported to be involved in the regulation of H19 expression (32). Loss of CTCF binding, regional CpG methylation and spread of heterochromatin have been shown in different disorders such as myotonic dystrophy caused by expanded CTG repeats (Cho et al. 2005). We evaluated in both sides of paravertebral muscles the level of CTCF occupancy in ICR and found reduced CTCF occupancy in the concave side. On the other hand, CpG methylation level in the promoter region of H19 showed no significant difference. These data thus suggest that CTCF occupancy changes in ICR of H19 might contribute to dysregulation of its expression in AIS. However, the mechanism of altered CTCF occupancy in two sides of muscle tissue in AIS remains unknown. There is increasing evidence that several environmental factors, such as dietary factors and sports, can reshape the epigenome and cause transcriptional changes in different tissues including skeletal muscle (Jacobsen et al. 2012; Feil and Fraga 2012). In one study, a 3-month exercise training was shown to induce modifications in DNA methylation that were associated to gene expression changes concordant with muscle functional and structural remodeling (Lindholm et al. 2014). Interestingly, some environmental factors have been reported to be involved in the etiopathogenesis and phenotypic expression of AIS (Burwell et al. 2011; Grivas et al. 2006; McMaster 2011). It has been reported that AIS to be positively associated with classical ballet training (Watanabe et al. 2017) and negatively associated with skating and horse riding classes (McMaster et al. 2015). Hence, further work is necessary to examine whether and how these factors alter CTCF occupancy of ICR of H19, particularly in the context of AIS.

The mRNA of MSTN gene showed significantly higher expressions in concave-sided muscle tissue in the first cohort (*n* = 10). But the difference was not significant in the second cohort (*n* = 50, Additional file 1: Figure S5). Myostatin, encoded by MSTN, is predominantly expressed in skeletal muscles and is a potent negative regulator of muscle growth and development (Rodriguez et al. 2014). It has been demonstrated that the muscle volumetric imbalance occur in all of the levels of the vertebrae involved in the major curve of AIS with larger muscle volume on the convex side (Jiang et al. 2017). Myostatin inactivation can induce skeletal muscle hypertrophy, while its overexpression or systemic administration causes muscle atrophy (Rodriguez et al. 2014; Sharma et al. 2015b). In our study, no difference expression of MSTN in AIS patients suggests the existence of additional regulatory mechanisms of muscle mass. In addition, KEGG enrichment analysis of DEGs showed genes involved in pathways of glycolysis/gluconeogenesis, adipocytokine signaling pathway, pyruvate metabolism and vascular smooth muscle contraction may be associated with the imbalance of paravertebral muscle in AIS patients. It is worthwhile to note that MYH3 expression showed imbalance in RNA-seq. MYH3 encodes embryonic myosin and its mutations contribute to spondylocarpotarsal synostosis (SCT), which is a skeletal disorder characterized by fused vertebrae (including scoliosis and lordosis) and fused carpal and tarsal joints (Cameron-Christie et al. 2018). And it is reported that MYH3 SCT mutations may influence the mechanics of contractility in muscle fibers between the neural arches and lead to SCT (Zieba et al. 2017). Whether the asymmetric expression of MYH3 in convex/concave paravertebral muscles is involved in the etiology of AIS should be further validated.

## Conclusion

We provide evidence that H19 and ADIPOQ are expressed inconsistently in paravertebral muscles, and more importantly, lower H19 levels and higher ADIPOQ levels in concave-sided muscle tissues positively correlate with spinal curve and age at initiation. These data suggest an important role of H19 and ADIPOQ in the onset or progression of scoliosis. Our results imply AIS may be a disease of genetically predisposed individuals with some environmental stress, which influence the epigenome and transcriptional changes in paravertebral muscles, overwhelm the stability of the spine and result in scoliosis, which can expand our etiologic understanding of this disease.

## Additional file

Additional file 1: **Table S1.** Clinical characteristics of enrolled AIS patients. **Table S2.** Sequence of RNA and DNA Oligonucleotides. **Table S3.** Differentially expressed genes (DEG) from RNA-seq. **Table S4.** The miR-675-5p potential target sites in ADIPOQ transcript according to the RNA22 software. **Figure S1.** Representative radiographic data of the CS patients enrolled in this study. **Figure S2.** Similar muscle tissue rate biopsy in each side of AIS patients. **Figure S3.** Expression difference of H19 or ADIPOQ mRNA in paravertebral muscles of AIS patients is not associated with age at menarche and BMI. **Figure S4.** The relative expression of miR-675-3p in concave/convex sided paravertebral muscles and the correlation of the expression level between miR-675-3p and miR-675-5p. **Figure S5.** The relative expression of MSTN mRNA in a larger AIS cohort. **Figure S6.** CpG methylation in the promoter region of H19 showed no difference between two sides of AIS patients. **Figure S7.** Fatty infiltration imbalance of deep paravertebral muscles in AIS patients. (DOCX 4564 kb)

## Abbreviations

AIS: Adolescent idiopathic scoliosis
CCTF: CCCTC-binding factor
CS: Congenital scoliosis
DEG: Differentially expressed genes
ICR: Imprinting control region
SNPs: Single nucleotide polymorphisms
