# Supplementary material for: Asymmetric expression of H19 and ADIPOQ in concave/convex paravertebral muscles is associated with severe adolescent idiopathic scoliosis
Source: Mol Med. 2018 Sep 18;24:48. doi: 10.1186/s10020-018-0049-y (PMC6145194; doi:10.1186/s10020-018-0049-y)

Additional file 1

Table S1. Clinical characteristics of enrolled AIS patients

|  | Discovery set (RNA-seq) (n=5) | Validation set (n=60) |
| --- | --- | --- |
| Sex, n (%) |  |  |
| Male | 0 (0) | 11 (18) |
| Female | 5 (100) | 49 (82) |
| Age at initiation, yrs, mean ± SD | 11.40 ± 1.14 | 11.52 ± 1.00 |
| Age at menarche, yrs, mean ± SD | 12.00 ± 1.00 | 12.84 ± 1.03 |
| Age at surgery, yrs, mean ± SD | 14.20 ± 1.92 | 15.25 ± 2.64 |
| Lenke classification, n (%) |  |  |
| 1 | 3 (60) | 34 (57) |
| 2 | 0 (0) | 3 (5) |
| 3 | 1 (20) | 15 (25) |
| 4 | 1 (20) | 8 (13) |
| Spinal curve, °, mean ± SD | 56.80 ± 6.06 | 54.48 ± 10.09 |
| Body mass index, Kg/m^2^, mean ± SD | 20.00 ± 1.58 | 21.30 ± 2.35 |

Table S2. Sequence of RNA and DNA Oligonucleotides

| Name | Sense Strand/Sense Primer (5’-3’) | | Antisense Strand/Antisense Primer (5’-3’) |
| --- | --- | --- | --- |
| **Primers for qRT-PCR** | | |  |
| H19 | GCACCTTGGACATCTGGAGT | | TTCTTTCCAGCCCTAGCTCA |
| PCK1 | ATGCCGATCTTTGACAGAGG | | GAGAAAGCGTTCAATGCCAG |
| FABP4 | TGATGATCATGTTAGGTTTGGC | | TGGAAACTTGTCTCCAGTGAA |
| SCD1 | GCAGCCGAGCTTTGTAAGAG | | GTTCTACACCTGGCTTTGGG |
| PLIN1 | AGGTCTTCTGGAAGCATTCG | | ACCTTGCTGGATGGAGACC |
| ADIPOQ | TAGAACAGCTCCCAGCAACA | | CCATCTCCTCCTCACTTCCA |
| MSTN | AAGACCAAAATCCCTTCTGGA | | CTGTAACCTTCCCAGGACCA |
| 18s | GCAATTATTTCCCATGAACGA | | ATCAACGCGAGCTTATGACC |
| miR-675-5p | GTCGTATCCAGTGCAGGGTCCGAGGTATTCGCACTGGATACGACCACTGTG | | |
| **RT primers** | | | |
| miR-675-5p | GTGCTGGTGCGGAGAGG | | GTGCAGGGTCCGAGGT |
| miR-675-3p RT primer | GTCGTATCCAGTGCAGGGTCCGAGGTATTCGCACTGGATACGACACTGAG | | |
| miR-675-3p | TATGCTTGTTCTCGTCTCTGTGTC | TATGCTTGTTCTCGTCTCTGTGTC | |
| U6 | CTCGCTTCGGCAGCACA | | AACGCTTCACGAATTTGCGT |
| **Mimics** |  | |  |
| miR-675-5p mimic | UGGUGCGGAGAGGGCCCACAGUG | | CACUGUGGGCCCUCUCCGCACCA |
| **Inhibitors** |  | |  |
| miR-675-5p inhibitor | CACUGUGGGCCCUCUCCGCACCA | |  |

Abbreviations: PCK1, Phosphoenolpyruvate Carboxykinase 1; FABP4, Fatty Acid Binding Protein 4; SCD1, Stearoyl-CoA Desaturase-1; PLIN1, Perilipin 1; ADIPOQ, adiponectin; MSTN, Myostatin

Table S3. Differentially expressed genes (DEG) from RNA-seq

Figure S1. Representative radiographic data of the CS patients enrolled in this study


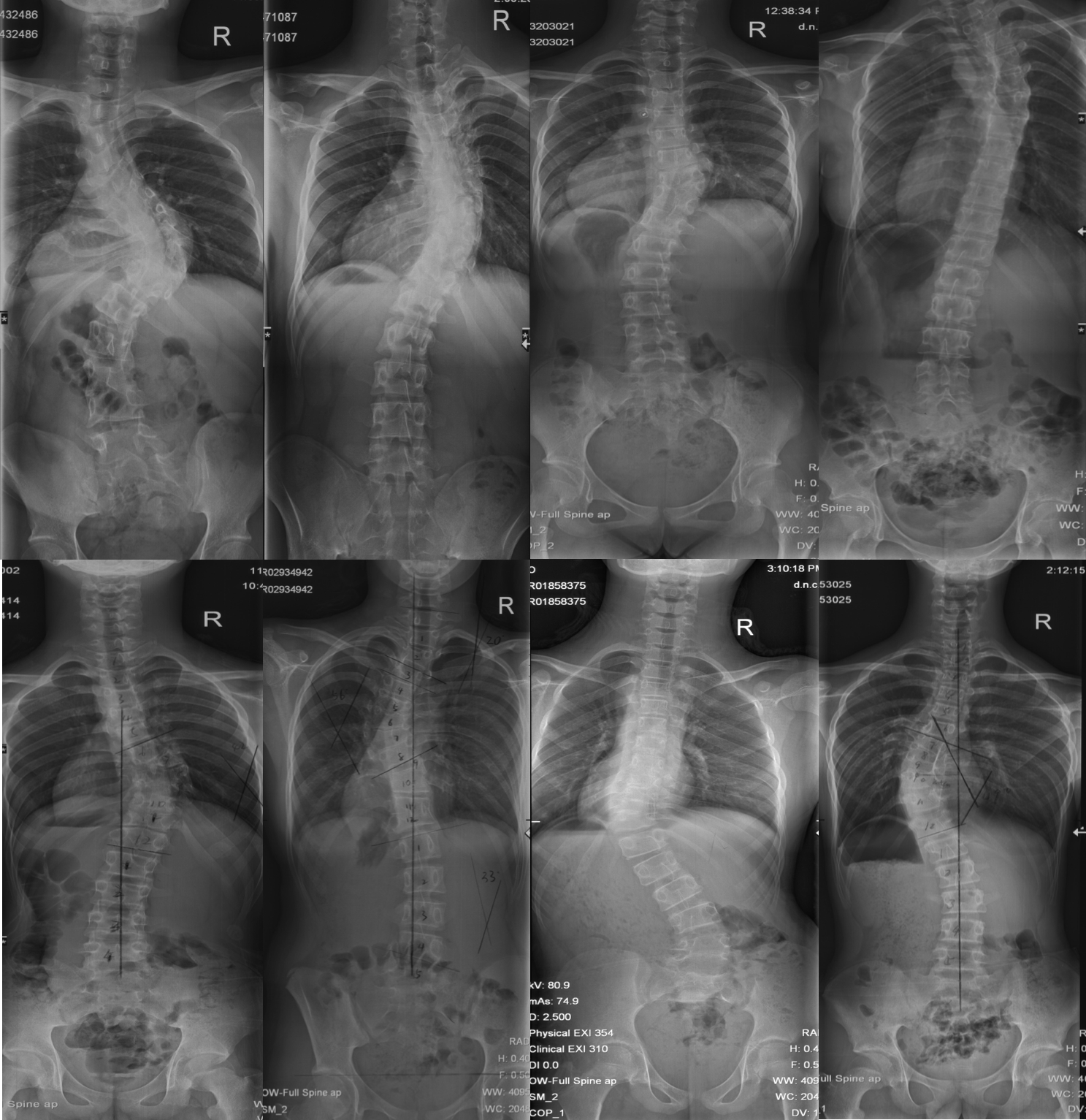


Figure S2. Similar muscle tissue rate biopsy in each side of AIS patients

Paravertebral muscles were obtained during the fusion surgery from AIS patients (n=10). Tissue samples were processed for assessment of expression of adipocytes (PPARγ, C/EBPα) and myocytes (Myog, MHC) markers, as described in Materials and Methods. Results are expressed as mean ± SEM.


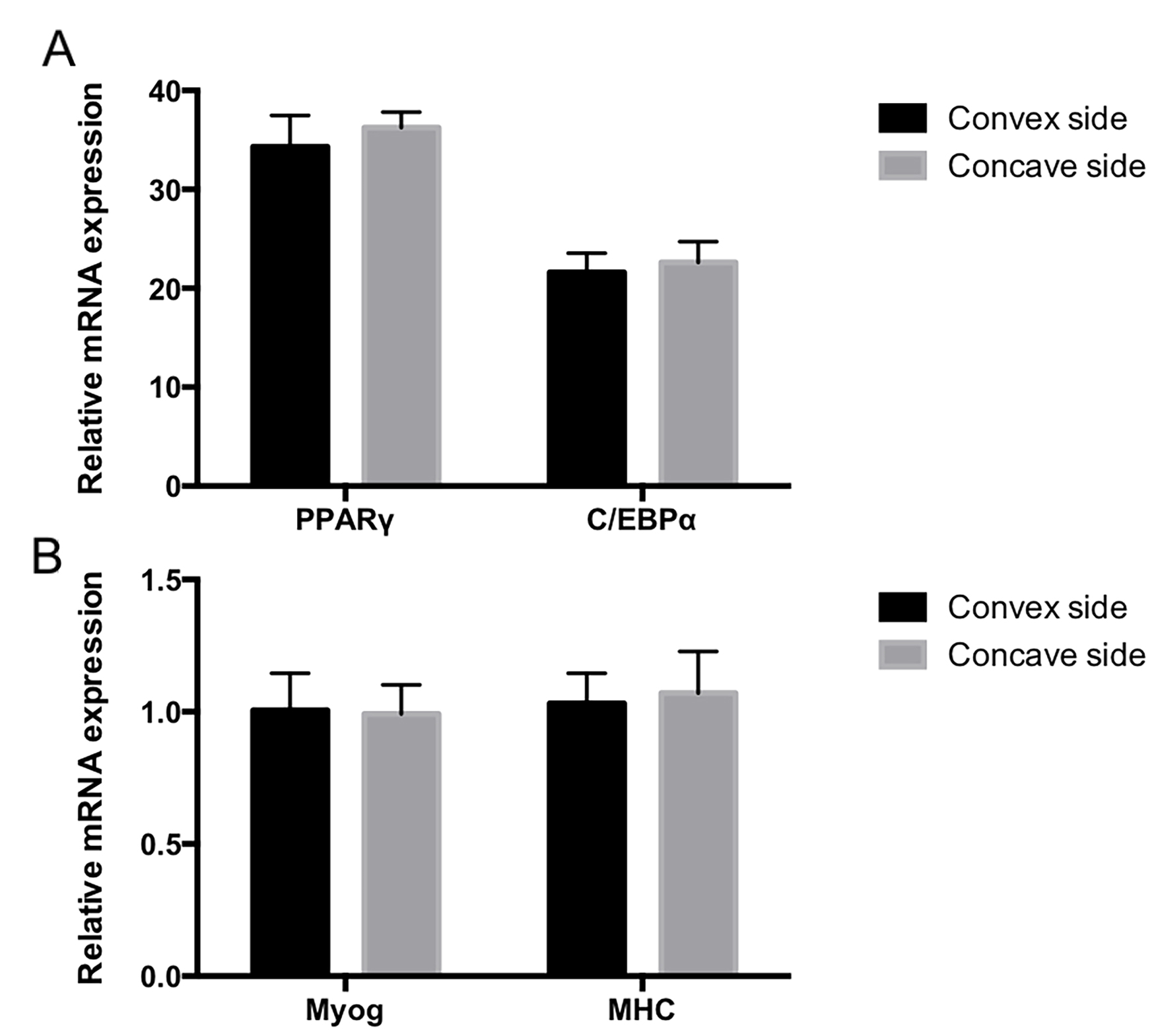


Figure S3. Expression difference of H19 or ADIPOQ mRNA in paravertebral muscles of AIS patients is not associated with age at menarche and BMI.

The relative expression difference of studied genes was calculated as the relative expression of one gene in concave sided muscle minus that in convex sided. For (A), Heat map showed that the expression difference of H19 or ADIPOQ mRNA in AIS patients (n=60) was not correlated with patients’ age at menarche. For (B), correlation analysis identified that the relative expression difference of H19 in AIS patients (n=60) was not correlated with BMI. For (C), the relative expression difference of ADIPOQ mRNA in AIS patients (n=60) was not correlated with BMI.


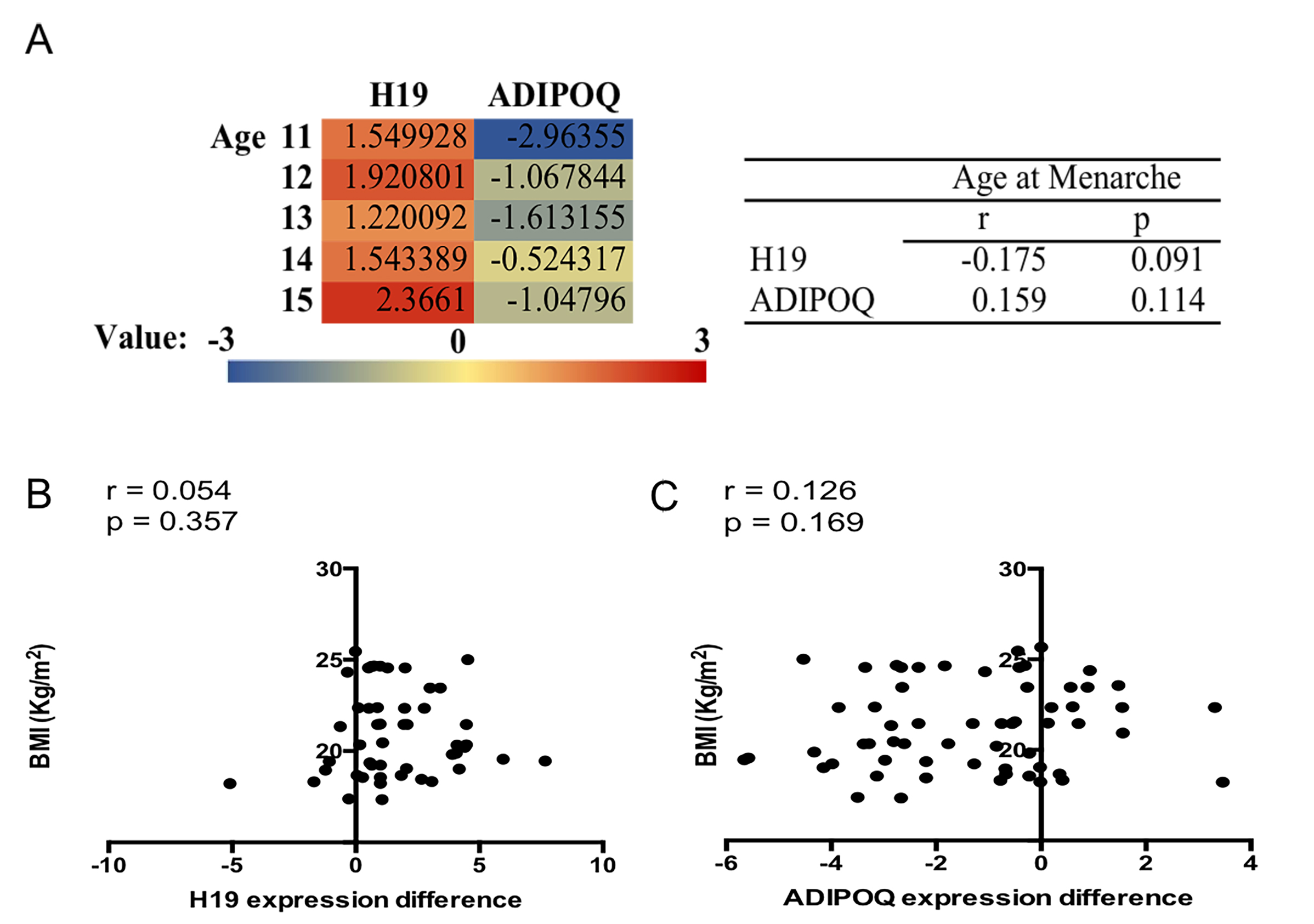


Figure S4. The relative expression of miR-675-3p in concave/convex sided paravertebral muscles and the correlation of the expression level between miR-675-3p and miR-675-5p

(A) Tissue samples were processed for assessment of expression of miR-675-3p as described in Materials and Methods. miR-675-3p showed a significantly higher expression in convex-sided muscle tissue in AIS patients (n=60). Results are expressed as mean ± SEM, and the relative expression of the studied genes are represented as fold change related to the convex side. (B) Correlation analysis identified that the expression level of miR-675-5p in muscle tissues was positively correlated with that of miR-675-3p in AIS patients (n=60).
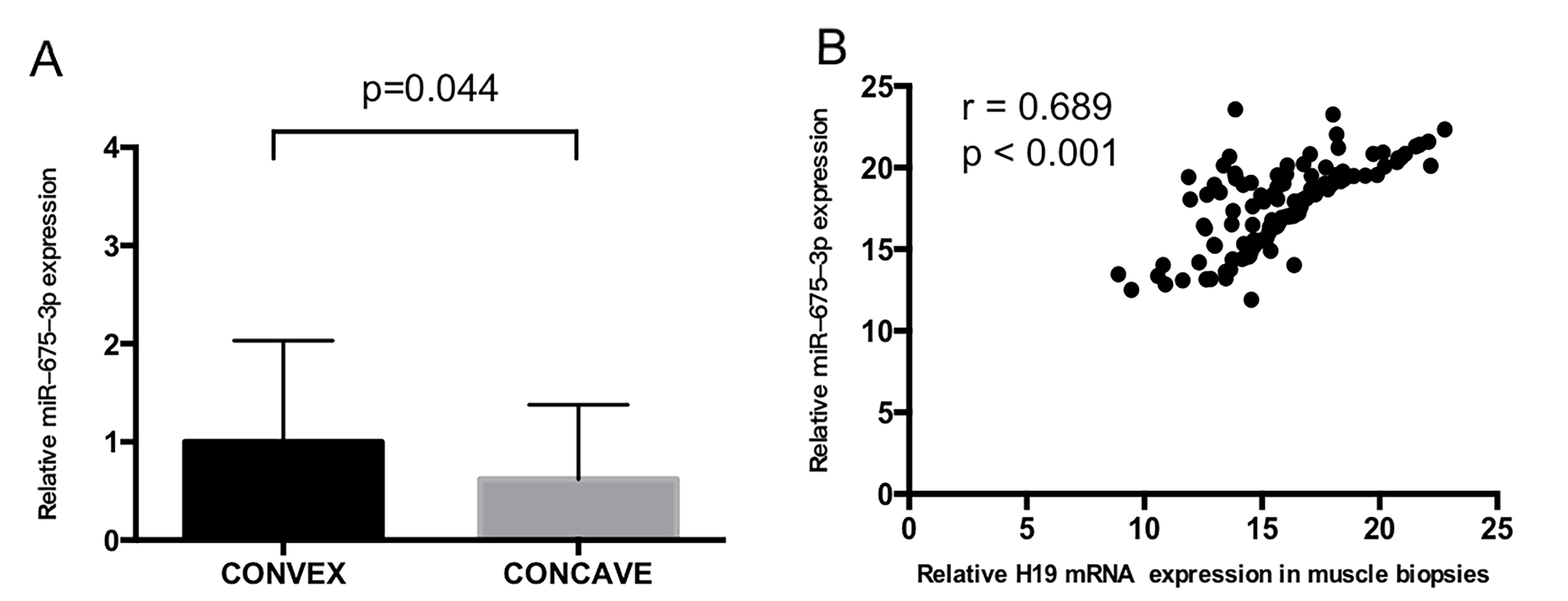


Table S4. The miR-675-5p potential target sites in ADIPOQ transcript according to the RNA22 software

| miRNA | Leftmost position of predicted targeted site | cDNA region | Folding energy (Kcal/mol) | Predicted target site | base pairs in putative heteroduplex | Span of target |
| --- | --- | --- | --- | --- | --- | --- |
| miR-675-5p | 477 | 3'UTR | -14.10 | CTCTGTTTCCCACCTCACCT | 13 | 20 |
| miR-675-5p | 905 | 3'UTR | -13.50 | CACACATGGCTAATTTTTGTATTT | 10 | 24 |
| miR-675-5p | 2371 | 3'UTR | -14.70 | CTCTCGTATCCCCAAGCCACACCA | 15 | 24 |
| **miR-675-5p** | **3403** | **3'UTR** | **-17.70** | **AACTACAGGCTCATGCCACTGCGCCC** | **13** | **26** |

The bold item indicates the predicted target site of maximum likelihood.

Figure S5. The relative expression of MSTN mRNA in a larger AIS cohort

Paravertebral muscles were obtained during the fusion surgery from 60 AIS patients. Tissue samples were processed for assessment of expression of MSTN as described in Materials and Methods. Results are expressed as mean ± SEM.


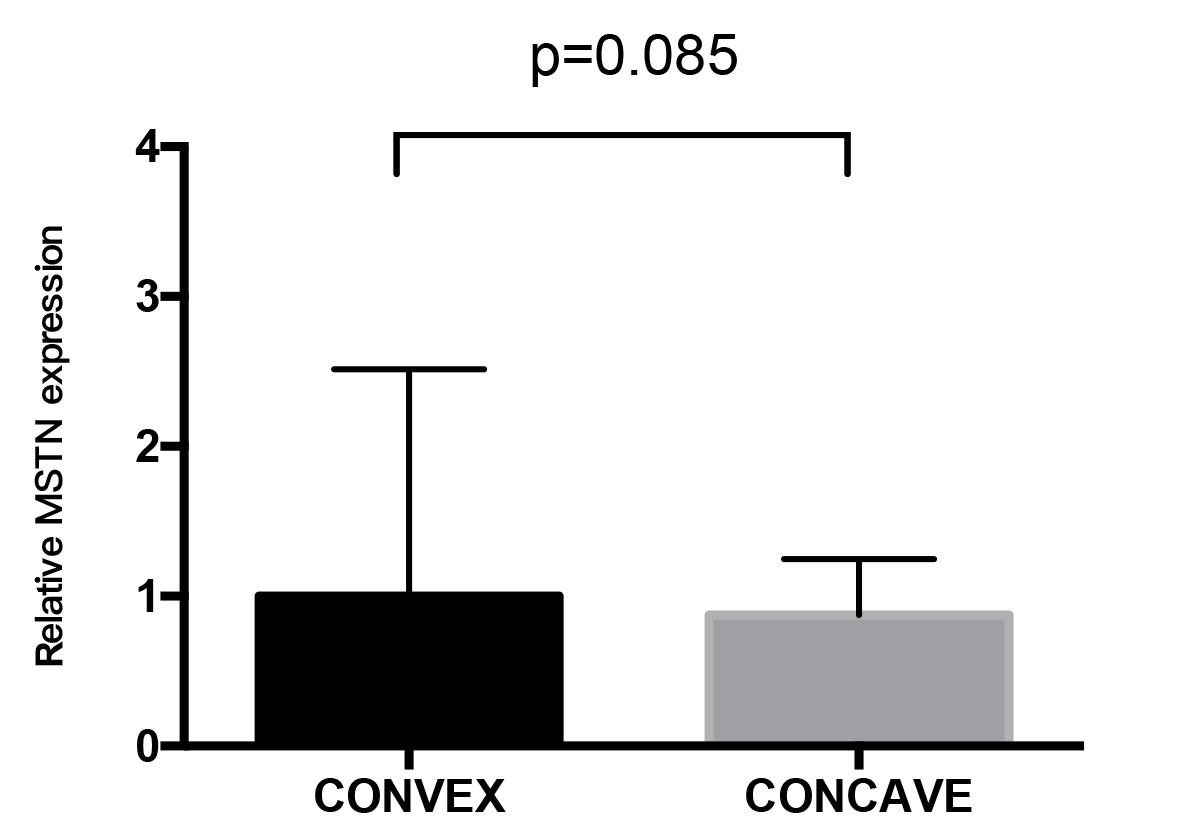


Figure S6. CpG methylation in the promoter region of H19 showed no difference between two sides of AIS patients

CpG methylation levels in the promoter region of H19 in 5 pairs of paravertebral muscle obtained from 5 AIS patients were assessed by methylation sequencing.


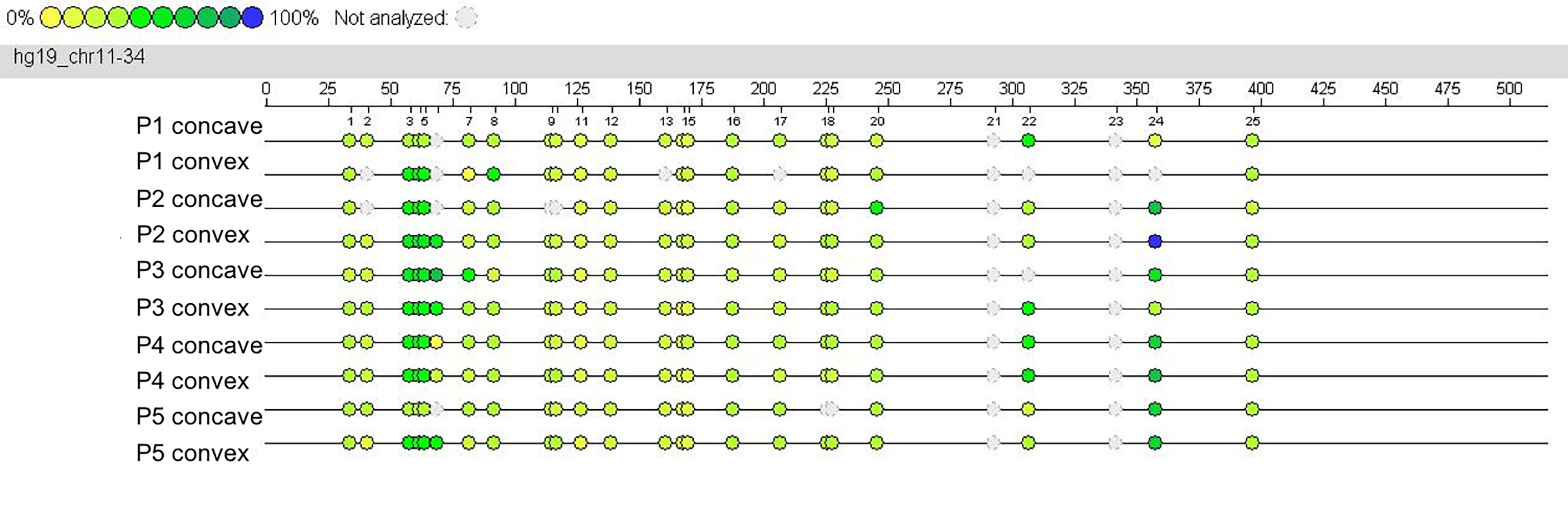


Figure S7. Fatty infiltration imbalance of deep paravertebral muscles in AIS patients

(A)Images of Oil red O staining in paravertebral muscle of AIS patients. Scale bar, 20μm. (B) Histograms show quantification of Oil red O staining by spectrophotometry. Results are expressed as mean ± SEM, and the relative expression of the studied genes are represented as fold change related to the convex side, *, P < 0.05 versus convex; **, P < 0.01 versus convex.


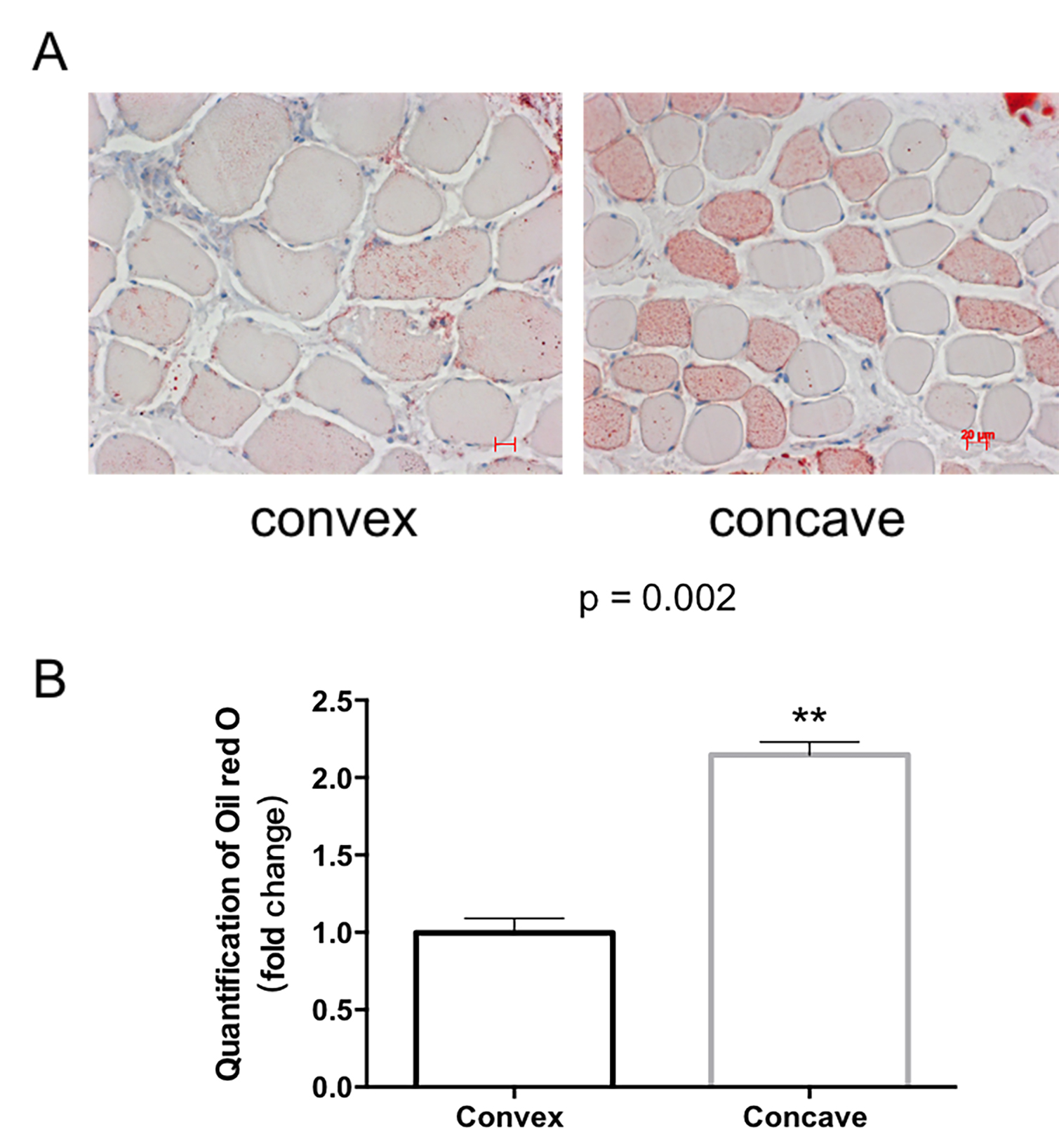

Supplement: Supplementary file 1 — Table S1. Clinical characteristics of enrolled AIS patients. Table S2. Sequence of RNA and DNA Oligonucleotides. Table S3. Differentially expressed genes (DEG) from RNA-seq. Table S4. The miR-675-5p potential target sites in ADIPOQ transcript according to the RNA22 software. Figure S1. Representative radiographic data of the CS patients enrolled in this study. Figure S2. Similar muscle tissue rate biopsy in each side of AIS patients. Figure S3. Expression difference of H19 or ADIPOQ mRNA in paravertebral muscles of AIS patients is not associated with age at menarche and BMI. Figure S4. The relative expression of miR-675-3p in concave/convex sided paravertebral muscles and the correlation of the expression level between miR-675-3p and miR-675-5p. Figure S5. The relative expression of MSTN mRNA in a larger AIS cohort. Figure S6. CpG methylation in the promoter region of H19 showed no difference between two sides of AIS patients. Figure S7. Fatty infiltration imbalance of deep paravertebral muscles in AIS patients. (DOCX 4564 kb) [file 10020_2018_49_MOESM1_ESM.docx]
